# The Battle of the Pneumonia Predictors: A Comprehensive Meta-Analysis Comparing the Pneumonia Severity Index (PSI) and the CURB-65 Score in Predicting Mortality and the Need for ICU Support

**DOI:** 10.7759/cureus.42672

**Published:** 2023-07-29

**Authors:** Hany A Zaki, Baha Hamdi Alkahlout, Eman Shaban, Eslam Hussein Mohamed, Kaleem Basharat, Wael Abdelrehem Elnabawy Elsayed, Aftab Azad

**Affiliations:** 1 Emergency Medicine, Hamad Medical Corporation, Doha, QAT; 2 Cardiology, Al Jufairi Diagnosis and Treatment, Doha, QAT

**Keywords:** psi criteria for pneumonia, methodological quality assessment, prognostic scoring methods, community-acquired pneumonia, systematic review and meta analysis, icu, mortality, curb 65, pneumonia severity index

## Abstract

The CURB-65 (confusion, uremia, respiratory rate, blood pressure, age ≥ 65 years) score and the pneumonia severity index (PSI) are widely used and recommended in predicting 30-day mortality and the need for intensive care support in community-acquired pneumonia. This study aims to compare the performance of these two severity scores in both mortality prediction and the need for intensive care support. A systematic review and meta-analysis was carried out, following the PRISMA (Preferred Reporting Items for Systematic Reviews and Meta-Analysis) 2020 guidelines, and PubMed, Scopus, ScienceDirect, and Google Scholar were searched for articles published from 2012 to 2022. The reference lists of the included studies were also searched to retrieve possible additional studies. Twenty-five studies reporting prognostic information for CURB 65 and PSI were identified. ReviewManager (RevMan) 5.4.1 was used to produce risk ratios, and a random effects model was used to pool them. Both PSI and CURB-65 showed a high strength in identifying high-risk patients. However, CURB-65 was slightly better in early mortality prediction and had more sensitivity (96.7%) and specificity (89.3%) in predicting admission to intensive care support. Thus, CURB-65 seems to be the preferred tool in predicting mortality and the need for admission into intensive care support.

## Introduction and background

The CURB-65 (confusion, uremia, respiratory rate, blood pressure, age ≥ 65 years) score and the pneumonia severity index (PSI) are widely used in predicting 30-day mortality and the need for intensive care support in community-acquired pneumonia (CAP) [1]. As optimal care necessitates quick diagnosis of very ill patients and proper emphasis on hospital admission and ICU admission, prognostic scoring methods for CAP were established to evaluate the severity of the disease and categorize patients based on mortality risk [2].

Severity assessment methods have been created to direct treatment locations for patients with CAP and, in particular, identify individuals whose illness may be safely treated at home [3]. Moreover, the discovery of coronavirus disease 2019 (COVID-19) led to the use of these severity scores in stratifying COVID-19 patients into low-risk or high-risk at the time of hospital admission [4,5]. The PSI and CURB-65 are the two most widely used of these tools. Both products were created using statistical analysis of characteristics linked to 30-day mortality [3,6]. Such characteristics are translated into a severity score representing the patient's mortality risk and can be used to determine whether inpatient or outpatient care is best. Although 30-day mortality is undoubtedly significant, most CAP patients who pass away are older people with multiple comorbidities [7]. These challenges may be overcome by offering a reliable, validated classification of patients into low, middle, and high-risk groups based on severity scores.

The PSI, also known as the pneumonia Patient Outcomes Research Team (PORT) score, has been the subject of the most research. It was created in 1997 due to a study involving more than 50 000 CAP patients [8]. Patients were divided into five risk categories (I-V) using a 20-point scoring system based on their percentage risk of passing away within 30 days, as presented in Table 1.

**Table 1:** The 20-point scoring system

Class I (low risk)	Physical examination findings, no comorbidities or laboratory findings
Class II (low risk)	≤70 points
Class III (low risk)	71–90 points.
Class IV (moderate risk)	91–130 points
Class V (high risk)	>130 total points

Patients are then monitored according to their PSI/PORT results. Outpatient care is provided for patients with scores below 70. Patients who score between 71 and 90 may get outpatient care or be admitted for observation. Patients who score more than 90 must be admitted for optimal care, and those who score more than 130 should receive ICU therapy for the best results. The PSI has been used successfully in clinical practice to increase the use of outpatient treatment in CAP, and it is recommended by various national and international guidelines [9]. However, the PSI has its limitations. It is challenging to apply in a crowded emergency department due to the high number of factors which strongly weigh age and co-morbid diseases. Later, the Infectious Diseases Society of America advised using the PSI scoring system as a predictor for patients with community-acquired pneumonia [8].

The CURB-65 score, which gives one point each for confusion, urea >7 mM/L (19 mg/dL), respiration rate ≥ 30/min, systolic blood pressure < 90 mmHg, and/or diastolic blood pressure ≤ 60 mmHg, and age ≥ 65 yr, was developed by an international study carried out in Europe (Table 2) [10].

**Table 2:** CURB-65 score CURB-65: confusion, uremia, respiratory rate, blood pressure, age ≥ 65 years

Variable	Value
Confusion	Mental Test Score ≤ 8, new disorientation in person, place or time
Urea	>7 mmol/L
Respiratory rate	≥30/min
Blood pressure	Systolic < 90 mmHg, and/or diastolic ≤ 60 mmHg
Age	≥ 65 years

Since there are only five variables and one point is given for each, this score is much simpler to understand and apply than the PSI. To calculate the score, each parameter is worth one point, and 0 to 5 is the possible score. Patients with CURB-65 scores of 3 to 5 have a higher mortality risk than those scoring between 0 and 2 [9].

In addition to the PSI, CURB65 is now recommended by other national and international recommendations due to its widespread adoption. However, CURB-65 too has limitations. For instance, by dividing patients into only two categories (severe or non-severe), it fails to identify individuals with a low risk of mortality who would be good candidates for early hospital departure or home treatment. The CRB-65, a similar instrument not detecting blood urea, might also be applied in the community. In Europe, especially for hospitalized patients, the CRB-65 score is widely used and recommended for outpatient usage without monitoring blood urea [11].

Given patients’ low quality of life and prognosis, rigorous treatment in the intensive critical care unit is sometimes viewed as inappropriate when such patients are admitted to the hospital [12]. As a result, methods for forecasting mortality are more accurate than determining which patients will benefit from admission to the critical care unit.

Clinicians might exaggerate and understate the seriousness of CAP, making it challenging to determine which patients must be sent to the critical care unit. A significant part of healthcare costs is spent on patients admitted to the CCU [12]. Early identification of such patients could lead to better results, fewer incorrect non-admissions, and possibly shorter ICU stays. In addition, ICU admission criteria vary between countries and hospitals. Conflicting findings have been reported in studies comparing different scoring systems, with some believing the PSI to be superior [13] and others finding it has no advantage over CURB-65 [14]. According to studies using CRB-65, this more straightforward approach may be similar to PSI and CURB-65 for predicting 30-day mortality.

There are several articles on prognostic scales for various comorbidities. However, it is still unclear which one is better at predicting severity in terms of mortality and the requirement for ICU admission in hospitalized patients. This review was conducted to compare PSI and CURB-65 in the prediction of mortality and the need for intensive care support, evaluate the compatibility of these scores with other comorbidities, and determine which scoring system is better in both.

## Review

Protocol and registration

This review followed the Preferred Reporting Items for Systematic Reviews and Meta-Analysis (PRISMA) 2020 guidelines [15]. We registered our protocol in the International Prospective Register of Systematic Reviews or PROSPERO.

Primary search

Three databases, PubMed/MEDLINE, Scopus, and ScienceDirect, were searched for relevant articles published between 2012 and October 2022 that presented information on mortality and the need for intensive care support concerning PSI and CURB-65 scores. A search string was developed for PubMed to conduct an e-databases search. The keyword search featured an all-text analysis to broaden the sensitivity of the search strategy. Our search was intended to capture articles presenting information on the comparative nature between PSI and CURB-65 scores on mortality and the need for intensive care support. The search strategies used for Scopus and ScienceDirect were slightly modified from the strategy used to search PubMed. The search string used in each database mentioned above is provided in Table 3.

**Table 3:** Search Strings

Database	Search string
PubMed	("Pneumonia severity index" OR PSI OR "PORT Score") AND (CURB-65 OR ‘CURB 65’ OR ‘C.U.R.B.65’ OR ‘C-U-R-B-65’) AND (mortality OR death OR fatality OR dying OR carnage) AND (ICU OR "intensive care unit" OR "intensive treatment unit" OR "emergency unit" OR "critical care unit" OR "intensive therapy unit")
Scopus	(("Pneumonia severity index" OR PSI OR "PORT Score") AND (CURB-65 OR ‘CURB 65’ OR ‘C.U.R.B.65’ OR ‘C-U-R-B-65’) AND (mortality OR death OR fatality OR dying OR carnage) AND (ICU OR "intensive care unit" OR "intensive treatment unit" OR "emergency unit" OR "critical care unit" OR "intensive therapy unit")) AND PUBYEAR >1999
ScienceDirect	("Pneumonia severity index" OR PSI OR "PORT Score") AND (CURB-65 OR "CURB 65") AND (mortality OR death) AND (ICU OR "intensive care unit")

Secondary search

In addition to the search conducted on the three databases, a direct search was done using the Google Scholar database. To allow the presentation of the most relevant results in the first pages, keywords representing mortality and Intensive care support (critical care unit, critical room, emergency unit, intensive treatment unit, loss of life, fatality, lethality, carnage, causality) were included in the search. The reference lists of the included studies were also searched for relevant additional articles.

Eligibility criteria

Two reviewers evaluated studies retrieved from the electronic databases using the inclusion and exclusion criteria. All studies had to meet the following pre-defined inclusion criteria: Original studies, including retrospective cohort studies, prospective cohort, and case-control studies; studies published in English; studies addressing mortality and the need for intensive unit support/admission, and presenting information for/of patients over 14 years of age. Studies that presented information of patients below 14 years of age were excluded. Studies that did not address mortality or the need for ICU but reported CURB 65 and PSI among CAP patients were excluded. Non-original articles like literature reviews, comments on published papers, letters to editors, conference papers, etc., non-peer reviewed, non-full text articles, and studies that did not report on any comparison between PSI and CURB 65 regarding mortality and ICU admission were excluded.

Review methods

Methodological Quality Assessment

The quality appraisal criteria used is a modification of the Newcastle-Ottawa Scale (NOS). The NOS was initially developed for cohort, case-control, and cross-sectional studies [16]. Hence, it had to be slightly modified to assess the quality of other study types. The criteria items and interpretation are described in Table 4. 

**Table 4:** Criteria items and interpretation of quality assessment via Newcastle-Ottawa scale

Criteria	Description
Selection	Representativeness of the exposed cohort. Selection of the non-exposed cohort. Ascertainment of exposure. Demonstration that outcome of interest was not present at the start of the study.
Comparability	Comparability of cohorts based on design or analysis.
Outcome	Assessment of outcome. Was follow-up long enough for outcomes to occur. Adequacy of follow-up of cohorts.

Data Extraction

Potentially eligible studies were individually screened using Zotero software. The selection featured a rigorous screening of titles, abstracts, and full texts. The full-text screening focused on presenting any form of data on the comparison between the PSI and CURB-65. After the selection of articles for inclusion, data was extracted into a predefined data descriptor table with the following fields: author, year of publication, country, study design (if presented), number of patients, age limit, mean age, mean duration of the hospital stay, mortality, sensitivity of PSI and CURB-65, and patients that were admitted in the ICU.

Synthesis of Results

Risk-of-bias appraisal was carried out through the robvis (Risk-Of-Bias VISualization) tool [17]. The heterogeneity was assessed across the included studies using a p-value and I^2^ statistics. A p-value having less than 0.10 was said to be evidence of heterogeneity. An I^2^ index between 50% and 70% was considered substantial heterogeneity, while an I^2^ value of more than 70% was regarded as the ultimate proof of study heterogeneity [18,19]. For calibration, the observed death rates from each trial were compared with the anticipated mortalities from the original derivation studies for PSI and CURB-65. The observed: and predicted risk ratios (RRs) are shown with 95% confidence intervals. The Review Manager (RevMan) Version 5.4 (2020; The Cochrane Collaboration) was used to produce RRs, and a random effects model was used to pool them. When the RR is more than 1, the observed mortality is more significant than predicted from the derivation study. An RR of less than 1 denotes that the number of deaths in the validation studies was lower than in the original derivation.

Results

**Figure 1:**
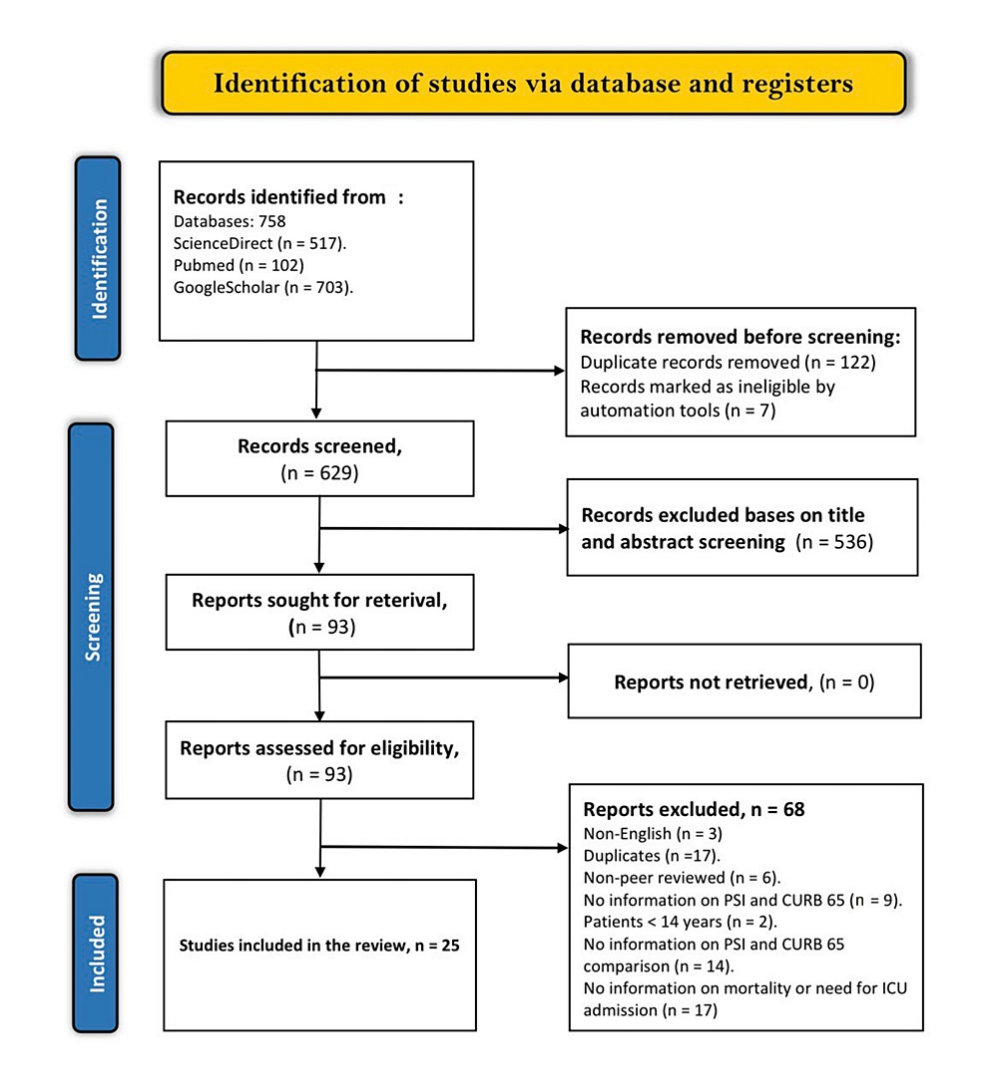
PRISMA flow chart illustrating the study selection procedure PRISMA: Preferred Reporting Items for Systematic Reviews and Meta-Analyses

Our search comparing PSI and CURB-65 retrieved 758 results from three primary databases. Of these, 122 articles were duplicates and were removed. Seven articles were removed because they were ineligible for inclusion as they did not contain any information on PSI and CURB-65 scores. The remaining articles were screened, and only 93 were sought for retrieval. We found no new additional articles for inclusion from Google Scholar and our search of the references of the included studies. The 93 articles were taken through the inclusion criteria, and only 25 studies were finally included in the review. Figure 1 represents the PRISMA flow chart summarizing the data screening procedure.

Quality Assessment Score

The quality assessment score according to the NOS is described in Table 5.

**Table 5:** Quality assessment score according to the Newcastle-Ottawa scale Selection: representativeness of studies (maximum score of 4); Comparability: comparability of studies based on the design or analysis or analysis (maximum score of 1); Outcome: assessment of outcome and follow-up (maximum score of 3); Studies with a total score of 6-8 were considered high quality while 1-5 were low-quality ones.

Study ID	Selection	Comparability	Outcome	Total score
Alavi-Moghaddam et al., 2013 [20]	4	1	3	8
Ozkan et al., 2020 [21]	4	1	3	8
Bahlis et al., 2021 [2]	4	1	3	8
Ronda et al., 2021 [22]	4	1	3	8
Feng et al., 2021 [3]	4	1	3	8
Neto et al., 2021 [6]	4	0	3	7
Estella, 2015 [23]	3	0	2	5
Demirel, 2018 [24]	1	1	3	5
Holten et al., 2020 [25]	4	1	3	8
Olivia et al., 2021 [26]	4	0	2	6
Cupurdija et al., 2015 [27]	4	1	3	8
Anurag and Preetam, 2021 [5]	4	1	3	8
Ranzani et al., 2017 [28]	4	1	2	7
Günaydın et al., 2019 [29]	4	0	3	7
Tsai et al., 2021 [30]	4	0	3	7
Wen et al., 2020 [31]	4	0	2	6
Putot et al., 2016 [7]	4	1	3	8
Wang et al., 2020 [32]	4	1	3	8
Bloom et al., 2019 [33]	4	0	1	5
Williams et al., 2018 [34]	4	0	3	7
Kim et al., 2013 [10]	4	1	2	7
Aydin et al., 2019 [35]	4	0	3	7
Akpınar et al., 2019 [36]	4	1	3	8
Ito et al., 2017 [37]	4	0	3	7
Lee et al., 2013 [38]	4	1	3	8

Risk-of-Bias Assessment

**Figure 2:**
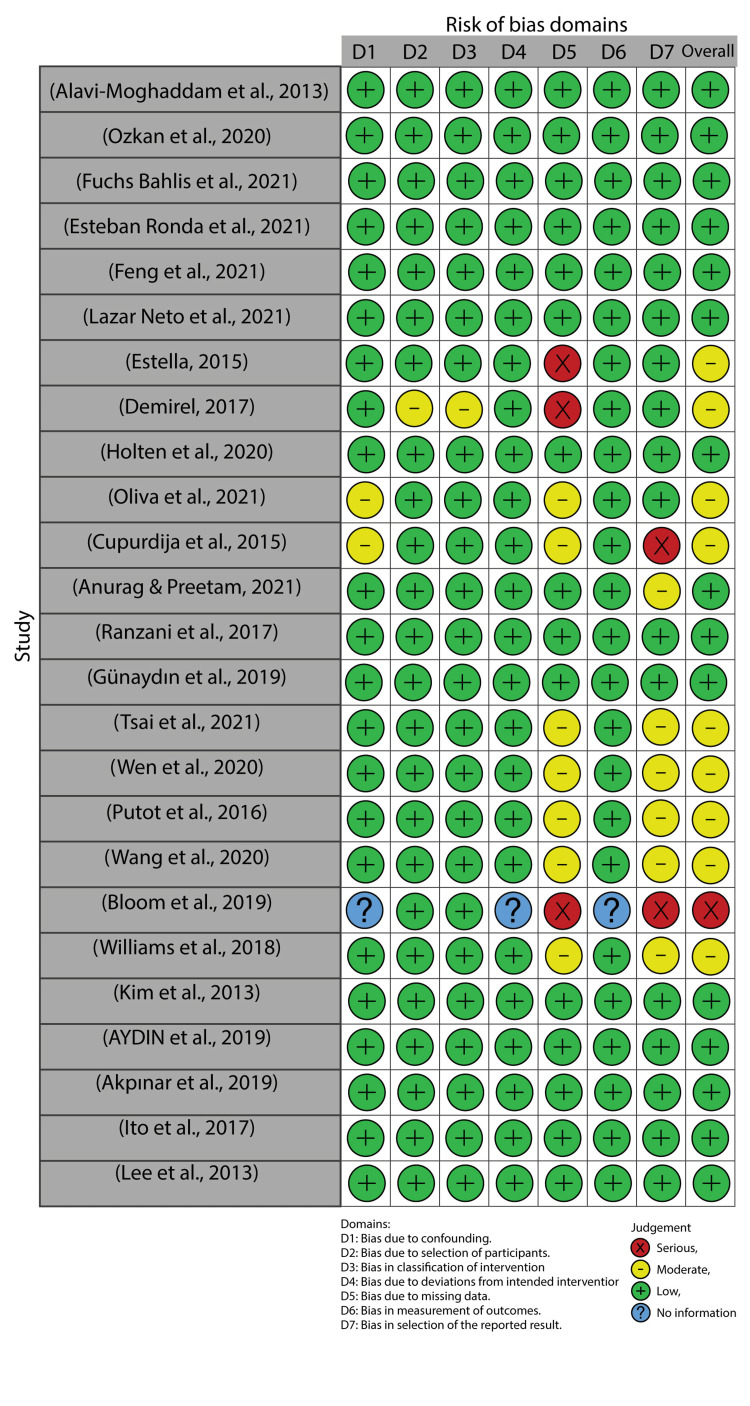
Traffic light plot Alavi-Moghaddam et al., 2013 [20], Ozkan et al., 2020 [21], Fuchs Bahlis et al., 2021 [2], Esteban Ronda et al., 2021 [22], Feng et al., 2021 [3], Lazar Neto et al., 2021 [6], Estella, 2015 [23], Demirel, 2018 [24], Holten et al., 2020 [25], Olivia et al., 2021 [26], Cupurdija et al., 2015 [27], Anurag and Preetam, 2021 [5], Ranzani et al., 2017 [28], Günaydın et al., 2019 [29], Tsai et al., 2021 [30], Wen et al., 2020 [31], Putot et al., 2016 [7], Wang et al., 2020 [32], Bloom et al., 2019 [33], Williams et al., 2018 [34], Kim et al., 2013 [10], Aydin et al., 2019 [35], Akpınar et al., 2019 [36], Ito et al., 2017 [37], Lee et al., 2013 [38]

**Figure 3:**
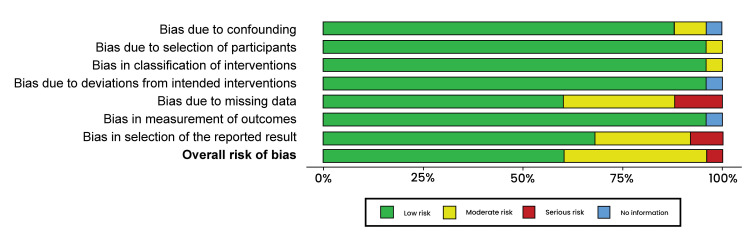
Risk of bias summary

Non-randomized intervention studies were evaluated for risk of bias. Traffic light plots and summary plots were then generated (Figures 2, 3).

Characteristics of Included Articles 

All the studies were published between 2012 and 2021 and reported data from national registries gathered in different timelines (Table 6). There were nine retrospective studies, five prospective observational cohort studies, three studies were comparative, two population-based observational studies, and six were a combination of three prospective observational studies. The included studies covered a broad scope of countries across the globe. Out of 25 studies, only one study [5] did not report data comparing PSI and CURB-65 in mortality, and 19 studies [2,3,6,10,20-23,25,26,28,29,32-38] reported data on the need for intensive care support. The size of the studies varied from 24 patients [23] to up to 6874 in the cohort study of Spain [28]. Reported mortality rates varied from 4.5% to 44.72%. A few studies employed in-hospital mortality as their primary end measure, but most studies used 30-day mortality. Hospitalized patients made up the vast bulk of the included research. Most studies had a mix of inpatients and patients managed in the community. Alavi-Moghaddam et al. provided exhaustive data on the mortality rate and need for intensive care support, specificity, and sensitivity of both severity scores, PSI and CURB-65 [20]. This study was thus used as the data referencing point.

**Table 6:** Characteristics of included studies

Author and year	Study Design	Study region	Population	Age	Mean age	Mean duration of hospital stay	Mortality rate	sensitivity (PSI/CURB 65)	Need for ICU (PSI/CURB 65)
Alavi-Moghaddam et al., 2013 [20]	Observational comparative study	Iran	200	>18 years	68	2-10.5 days	36 died	90%/96.7%	52 patients (specificity: 78.7%/89.3%)
Ozkan et al., 2020 [21]	Comparative study	Turkey	250	> 18 years	72.3	30 days	27 died	66.7%/88.9	80 patients
Fuchs Bahlis et al., 2021 [2]	Cohort Study	Brazil	304	≥ 14 years	67.1	7.2±7.4 days	47 died	3.7%/4.5%	89 patients
Esteban Ronda et al., 2021 [22]	Retrospective observation study	Spain	208	≥ 18 years	63	6-13 days	26 died	84.62%/88.46%	38 patients
Feng et al., 2021 [3]	Prospective cohort study	China	239	≥ 18 years	61.09	10 days	22 died	-	71 patients
Lazar Neto et al., 2021 [6]	Retrospective cohort study	Spain and Brazil	1363	≥18 years	61.05	7 days	320 died	59.9%/56.0%	646 patients
Estella, 2015 [23]	Retrospective observation study	Spain	24	unspecified	unspecified	unspecified	21.1%	CURB 65 scale of 1 (60%), 13.3% obtained 0 and 26.7% 2. PSI scale resulted class I in a 20%, class II 40%, 26.7% class IV, and 13.3% class V	19 patients
Demirel, 2018 [24]	Prospective cross-sectional study	Turkey	unspecified	unspecified	71±16.5	unspecified	21.8%	90.9%/90.9%	unspecified
Holten et al., 2020 [25]	Prospective cohort study	Norway	175	≥18 years	59	14 days	13 died	71%/58%	29 patients
Olivia et al., 2021 [26]	Retrospective single-center study	Italy	224	unspecified	unspecified	28 days	24 died	-	26 patients
Cupurdija et al., 2015 [27]	Prospective cohort study	Serbia	95	≥18 years	unspecified	14 days	5 died	22%/29%	unspecified
Anurag & Preetam, 2021 [5]	Retrospective observational study	India	122	≥11 years	44.16	14 days	-	47.6%/28.6%	unspecified
Ranzani et al., 2017 [28]	Cohort Study	Spain	6874	≥18 years	66.1	30 days	442 died	92%/78%	950 patients
Günaydın et al., 2019 [29]	Case-control study	Turkey	63	≥18 years	72.05	30 days	4 died	85.2%/82.4%	5 patients
Tsai et al., 2021 [30]	Retrospective cohort study	Australia	203	≥18 years	unspecified	30 days	13 died	-	unspecified
Wen et al., 2020 [31]	Retrospective observational study	China	223	≥18 years	unspecified	30 days	41 died	66%/85%	unspecified
Putot et al., 2016 [7]	Retrospective cohort study	France	217	≥75 years	unspecified	1 year	19.8%	66%/58%	unspecified
Wang et al., 2020 [32]	Retrospective observational study	China	123	unspecified	unspecified	180 days	55 died	8.1%/5.1%	68 patients
Bloom et al., 2019 [33]	Comparative study	Germany	276	≥60 years	unspecified	unspecified	unspecified	unspecified	11 patients
Williams et al., 2018 [34]	Prospective cohort study	Australia	618	unspecified	unspecified	30 days	12.1%	98%/94%	75 patients
Kim et al., 2013 [10]	Prospective cohort study	Korea	883	≥18 years	unspecified	30 days	40 died	4.5%/2.3%	80 patients
Aydin et al., 2019 [35]	Observational study	Turkey	159	≥18 years	66	28 days	58 died	8.6%/10.0%	72 patients
Akpınar et al., 2019 [36]	Prospective observational study	Brazil	155	≥18 years	72.7	30 days	unspecified	46.5%/36.1%	42 patients
Ito et al., 2017 [37]	Prospective cohort study	Japan	1834	≥15 years	73.5	30 days	122 died	24.9%/17.9	95 patients
Lee et al., 2013 [38]	Comparative study	Korea	208	>65 years	80	30 days	21.1%	40.0%/50.0%	55 patients

Results of Included Studies

Alavi-Moghaddam et al.’s study [20] was used to compare the data collected on the prediction of mortality and the need for ICU admission among patients from the other studies and as a data extraction guide for articles that generally focused on mortality. They established that the mortality was 18%, while the PSI and CURB 65 mortality/sensitivity predictions were 90% and 96.7%, respectively [20]. The mortality predictions were for the highest classes for both severity scores. These percentages showed that the patient's death was most likely. On the other hand, the prediction/specificity for ICU admission was 78.7% and 89.3%, respectively, for PSI and CURB 65 [20]. The CURB-65, however, seemed more specific in identifying the mortality risk in severe CAP. This study showed that 52 patients were admitted for critical care support. Eighteen studies were used in the meta-analysis to check the comparison of CURB 65 and PSI. The eighteen studies were further subdivided into two classes; the lowest class and the highest class. For PSI prediction, the lowest class was class I-II, while CURB 65 was class 0-1. As for the highest classes, PSI was class V, and CURB 65 was class 3-5. CURB-65 and PSI ratings both demonstrated a reasonable positive predictive value. 

**Figure 4:**
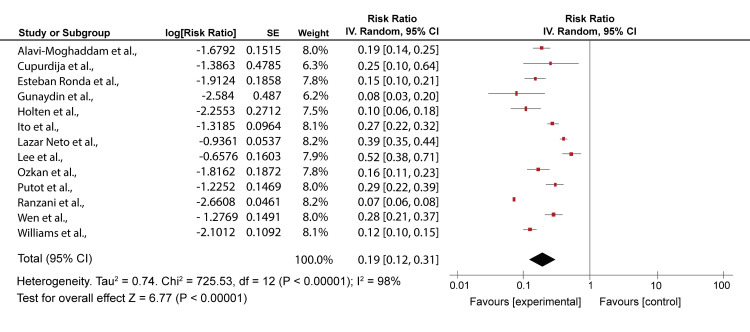
A forest plot of PSI scores risk ratios for class V PSI: Pneumonia Severity Index Alavi-Moghaddam et al., 2013 [20], Ozkan et al., 2020 [21], Esteban Ronda et al., 2021 [22], Lazar Neto et al., 2021 [6], Holten et al., 2020 [25], Cupurdija et al., 2015 [27], Ranzani et al., 2017 [28], Günaydın et al., 2019 [29], Wen et al., 2020 [31], Putot et al., 2016 [7], Williams et al., 2018 [34], Ito et al., 2017 [37], Lee et al., 2013 [38]

**Figure 5:**
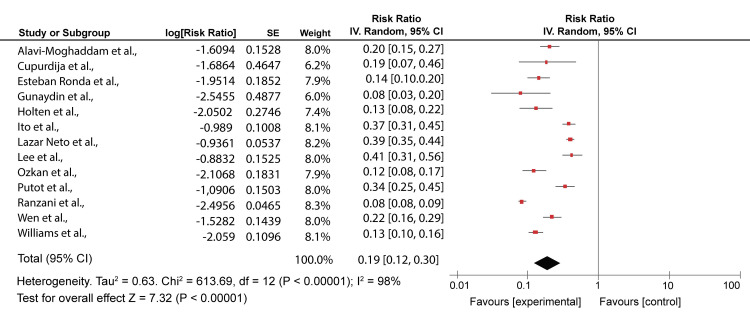
A forest plot of CURB-65 score risk ratios for class 0-3 Alavi-Moghaddam et al., 2013 [20], Ozkan et al., 2020 [21], Esteban Ronda et al., 2021 [22], Lazar Neto et al., 2021 [6], Holten et al., 2020 [25], Cupurdija et al., 2015 [27], Ranzani et al., 2017 [28], Günaydın et al., 2019 [29], Wen et al., 2020 [31], Putot et al., 2016 [7], Williams et al., 2018 [34], Ito et al., 2017 [37], Lee et al., 2013 [38] CURB-65: confusion, uremia, respiratory rate, blood pressure, age ≥ 65 years

A comparison was made for the highest classes for both severity scores in 13 studies [6,7,20-22,25,27-29,31,34,37,38]. Ozkan et al.’s [21] study had 66.7%/88.9, Alavi-Moghaddam et al. [20] 96.7%/90%, Esteban Ronda et al. [22] 84.62%/88.46%, Lazar Neto et al. [6] 59.9%/56.0%, Holten et al. [25] 71%/58%, Cupurdija et al. [27] 22%/29%, Ranzani et al. [28] 92%/78%, Günaydın et al. [29] 85.2%/82.4%), Wen et al. [31] 66%/85%, Putot et al. [7] 66%/58%, Williams et al. [34], 98%/94%, Ito et al. [37] 24.9%/17.9, and Lee et al. [38] 40.0%/50.0% for PSI and CURB-65 mortality predictions, respectively. The mortality rate for PSI class V ranged from 60% to 96%. This meant there was a high likelihood of the death of patients. Using CURB-65 for the same articles, class 3-5 prediction was almost similar with slight differences, as shown in Figures 4, 5.

CURB-65 had mortality rates ranging from 58% to 90.9%. This was similar to the PSI predictions. Both severity scores showed a high strength in identifying high-risk patients. However, CURB-65 accurately predicted 30-day mortality compared to the PSI score. Also, CURB-65 seemed more effective than PSI at identifying patients at the most significant risk [20]. Heterogeneity for class V and class 3-5 was high (I^2^ = 98%).

**Figure 6:**
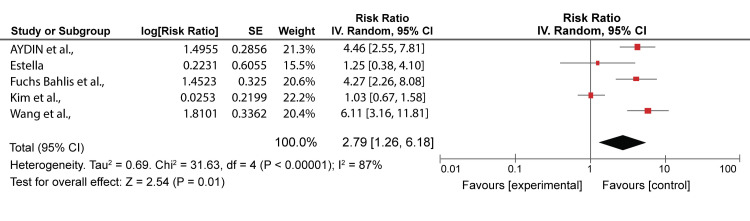
A forest plot of PSI risk ratios for the lowest class (class I -II) Fuchs Bahlis et al., 2021 [2], Estella, 2015 [23], Wang et al., 2020 [32], Kim et al., 2013 [10], Aydin et al., 2019 [35] PSI: pneumonia severity index

**Figure 7:**
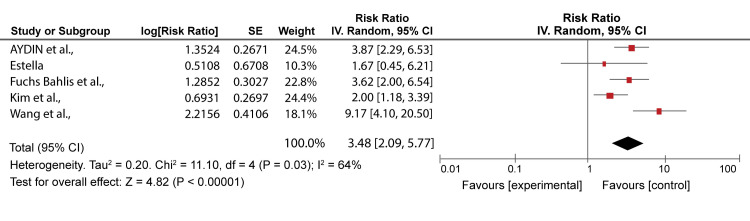
A forest plot of CURB-65 risk ratios for the lowest class (class 0-1) Fuchs Bahlis et al., 2021 [2], Estella, 2015 [23], Wang et al., 2020 [32], Kim et al., 2013 [10], Aydin et al., 2019 [35] CURB-65: confusion, uremia, respiratory rate, blood pressure, age ≥ 65 years

The remaining five articles [2,10,23,32,35] were compared for mortality rates in the lowest classes; class I-II and class 0-1 for PSI and CURB 65, respectively. Bahlis et al. [2] had 3.7%/4.5%, Estella [23] 20%/13.3%, Wang et al. [32] 8.1%/5.1%, Kim et al. [10] 4.5%/2.3%, and Aydin et al. [35] 8.6%/10.0% mortality rates by PSI and CURB 65 prediction, respectively. The range for mortality rate in PSI scores was 3.7-24.9%. The range showed that the lesser scores in PSI severity, the less likelihood of death of patients; however, CURB-65 can be a good severity score in the lower classes compared to PSI. Similarly, the range showed that the patients may be discharged from the hospital and get treatment from their homes. CURB-65 varied slightly compared to PSI, ranging from 2.3% to 17.9%. The PSI can better identify patients who can be safely discharged and managed at home compared to the CURB-65 score, which is less likely to be used [18]. The comparison is illustrated in Figures 6-7, which show similarities between CURB-65 and PSI. CURB-65 offers a straightforward technique for recognizing patients with high mortality risk and who might profit from early ICU admission [28]. Results in Figure 6 showed substantial heterogeneity (I^2^ = 87%), while results in Figure 7 showed moderate heterogeneity (I^2^ = 64%).

CURB-65 had more sensitivity (96.7%) and specificity (89.3%) in predicting admission to intensive care support, as seen in Table 6.

Discussion

It's interesting to note that additional comparisons of the PSI and CURB-65 instruments have highlighted significant variations in benefits and restrictions. The requirement to identify patients with low mortality risk served as the foundation for the creation and validation of PSI. Therefore, this instrument may not be accurate for choosing the location of therapy and may underestimate the severity of the illness, especially in young patients without concomitant illnesses [1]. According to this review, when comparing the PSI and CURB-65 tools in the same group, both were effective at predicting death and spotting low-risk patients. For the opportunity costs associated with missed productivity, the CURB-65 and PSI ratings both demonstrated a reasonable positive predictive value. However, we could not find statistically significant relationships between the direct expenses of CAP treatment and clinical severity ratings. The CURB-65, however, seemed more specific in identifying the mortality risk in severe CAP [39,40]. Many inpatient and outpatient CAP patients were assessed using the PSI and the CURB-65 in a different investigation. The CURB-65 accurately predicted 30-day mortality, the requirement for mechanical ventilation, and perhaps the need for hospital admission [41]. The time to clinical stability was also connected with the CURB-65 score. CURB-65 seems to be more effective than PSI at identifying patients who are at the most significant risk. As a result, it may be better suited to direct investigations and the administration of broad-spectrum antibiotic therapy (as recommended by the British Thoracic Society (BTS) guidelines when these actions are directed explicitly at high-risk patients [42]. The PSI was good in predicting death but not the necessity for ICU admission. The CURB-65 tool was reported to be more accurate than the PSI for this site-of-care choice in the study by Capelastegui et al. but the authors also noted that it could not predict the requirement for ICU admission [41].

A study by Dhawan et al. supported PSI as the best available predictor for nursing home-acquired pneumonia (NHAP), while CURB-65 was an alternative indicator [43]. PSI has also been seen as the best indicator in predicting different clinical outcomes in the elderly with CAP compared to CURB-65 [44]. PSI's predictive efficacy differs depending on the cause of CAP in adults. According to Rello, PSI is better than CURB-65 and more effective in cases of mild bacterial infection [45]. The systematic application of objective criteria to pneumonia patients' site-of-care decision-making is emerging as a breakthrough in patient management. The PSI can identify patients who can be safely discharged and managed at home. Still, it can also understate severity, especially in young patients with severe respiratory failure who don't have any co-morbid conditions [46]. Besides, CURB-65 offers a straightforward technique that can recognize individuals at a high mortality risk and who might profit from early ICU admission.

Overall test performance did not differ significantly across these scores, indicating that physicians may select the scoring system that is most appropriate for their particular circumstances. Even though the tests' overall accuracy was comparable, there were some differences in the performance traits between the scores. A more considerable positive predictive value shows that CURB-65/CRB-65 may be preferable for identifying high-risk patients. Still, a low negative likelihood ratio suggests that PSI may be superior for identifying low-risk patients. It is challenging to determine the therapeutic significance of these discrepancies, though. Therefore, it is crucial that these tools accurately forecast the desired outcome. Our meta-analysis shows that the severity scores predict the 30-day mortality from CAP scores using PSI and CURB-65 with characteristics of moderate-good performance. The PSI is considerably more complicated than the CURB-65 because it calls for measuring 20 separate parameters, with varying points given for each [8].

Limitations

First, the heterogeneity of the included studies severely constrained our meta-analysis. Different outcome measures, such as 30-day or in-hospital mortality, were utilized in numerous studies that included inpatients and outpatients. Different study strategies were employed, including prospective observational studies, retrospective studies, population-based observation, and comparative research. Additionally, some studies presented the mortality rate in percentages. Consequently, converting the percentages to numbers for meta-analysis could have contributed to errors due to rounding off the decimals to whole numbers. Furthermore, the mortality rate for people with mild CAP may be higher as it included deaths from non-infectious and unknown causes.

## Conclusions

This review attempted to provide a comparison of the PSI and CURB-65 on their abilities to predict mortality and the need for intensive care support. We formulated a research question and included relevant papers. Both narrative synthesis and meta-analysis were performed on the data taken from the included articles. Both severity scores have identical sensitivity, but the CURB-65 score was more specific than the PSI. Moreover, the CURB-65 score showed more sensitivity (89.3%) in ICU prediction than PSI (78.8%). It was seen that the two severity scores are the most preferred tools in the prediction of ICU admission and mortality. However, despite having comparable mortality, CURB-65 is highly preferred as it is easier to implement apart from everything else. Finally, even though these two scores are helpful tools, they cannot and should not be used in place of clinical judgment and medical examination. Ideally, the best strategy depends on doctors' experience and how well they apply their knowledge to the individual patients' scores to make the appropriate decision for ICU admission.
